# Influence of Melatonin Treatment on Cellular Mechanisms of Redox Adaptation in K562 Erythroleukemic Cells

**DOI:** 10.3390/genes13122337

**Published:** 2022-12-11

**Authors:** Flaviene Felix Torres, Victoria Simões Bernardo, Carla Peres de Paula, João Pedro Maia de Oliveira da Silva, Eduardo Alves de Almeida, Anderson Ferreira da Cunha, Danilo Grünig Humberto da Silva

**Affiliations:** 1 Department of Biology, Universidade Estadual Paulista (UNESP), São José do Rio Preto 15054-000, SP, Brazil; 2 Department of Genetics and Evolution, Universidade Federal de São Carlos (UFSCar), São Carlos 13565-905, SP, Brazil; 3 Department of Natural Sciences, Fundação Universidade Regional de Blumenau (FURB), Blumenau 89030-000, SC, Brazil; 4 Campus de Três Lagoas, Universidade Federal de Mato Grosso do Sul (CPTL/UFMS), Três Lagoas 79613-000, MS, Brazil

**Keywords:** N-[2-(5-methoxy-1H-indol-3-yl)ethyl], Nrf2, FoxO3, antioxidant therapy

## Abstract

Melatonin (MEL) presents well-documented pleiotropic actions against oxidative stress (OS), acting indirectly through activation of transcription factors, e.g., FoxO3 and Nrf2. Thus, this study aimed to investigate the possible modulating effects of MEL on the redox signaling pathways PI3K/AKT/FoxO3 and Keap1/Nrf2/ARE in K562 erythroleukemic cells subjected to OS induction. For this, the viability, and transcript levels of genes involved in redox adaptation were evaluated in K562 cells in different periods of erythroid differentiation: under OS induction by hydrogen peroxide (100 µM H_2_O_2_); treated with 1 nM (C1) and 1 mM (C2) MEL; and associated or not with stress induction. We observed a restoration of physiological levels of Nrf2 in both MEL concentrations under OS. The C1 was related to enhanced expression of antioxidant and proteasome genes through the Nrf2-ARE pathway, while C2 to the induction of FOXO3 expression, suggesting an involvement with apoptotic pathway, according to BIM transcript levels. The effects of MEL administration in these cells showed a period and dose-dependent pattern against induced-OS, with direct and indirect actions through different pathways of cellular adaptation, reinforcing the importance of this indolamine in the regulation of cellular homeostasis, being a promising therapeutic alternative for diseases that present an exacerbated OS.

## 1. Introduction

Oxidative stress (OS) poses a serious threat to the chemical integrity of biomolecules, such as lipids, proteins, and DNA, which often results in serious health problems [1,2]. Redox homeostasis has already been described as “the golden means of healthy living” and its regulation can occur through the control of enzymatic activities or at the transcriptional level through activation of different transcription factors (TFs) [3,4]. Among the TFs, Forkhead box O3 (FoxO3) plays indispensable functions on cell differentiation, autophagy, and apoptosis [5], besides redox adaption [3]. The transcriptional activity of this TF is mainly regulated through post-translational modifications (PTM) by the phosphatidylinositol 3 kinase/protein kinase B (PI3K/AKT) signaling pathway [3]. AKT phosphorylates FoxO3, breaking its bond to DNA and causing its translocation from the nucleus to the cytoplasm [6]. The translocation is performed by chaperone 14-3-3, which protects FoxO3 from being dephosphorylated during the process [7,8]. Mammalian sterile 20-like 1 (MST1) pathway is antagonistic to that of PI3K/AKT and when under oxidative stress, it is activated, enabling FoxO3 to start the transcription of target genes [7,9].

Another important TF regulated by the PI3K/AKT pathway is Nuclear Factor Erythroid-related Factor 2 (Nrf2) [10,11,12]. Under physiological oxidative conditions, Nrf2 is efficiently ubiquitinated by Keap1-Cul3 E3 ligase and rapidly degraded through the proteasome pathway. In contrast, when under oxidative stress, the complex containing Kelch-like ECH-associated protein 1 (Keap1) loses its ability to ubiquitinate Nrf2 [13], enabling this TF to bind to the antioxidant response element 2 (ARE) located in the regulatory regions of many cytoprotective enzyme genes [14], such as superoxide dismutase 1 (*SOD1*), catalase (*CAT*), glutathione peroxidase 1 (*GPX1*), peroxiredoxins 1, 2, 5, and 6 (*PRDX1*, *2*, *5* and *6*), the proteasome subunit beta type 5 and 6 (*PSMB5* and *6)* [15,16], and many other genes, detoxifying cells of all types of harmful substances [13]. Thus, due to the importance of redox homeostasis, the search for therapeutic alternatives that contribute to its maintenance is extremely valuable and promising for pathophysiological states related to chronic oxidative and inflammatory conditions. 

In this regard, melatonin (N-acetyl-5-methoxytryptamine) and its metabolites constitute a particularly efficient chemical family that offer protection against the harmful effects of oxidative stress [17]. Numerous studies have proven that melatonin has most of the desirable characteristics of good free radical depletors [17,18,19,20,21]. Both in vitro and in vivo, it acts as a broad-spectrum antioxidant, being able to act directly as an antioxidant, by interacting with free radicals and reactive oxygen and nitrogen species (ROS and RNS, respectively), inactivating them, or indirectly modulating activity and/or expression of the molecules, producing reactive species or of members of the cellular antioxidant system. Thus, it is able to act synergistically with the classic radical scavengers, making them more effective or stimulating the expression and, consequently, the activity of endogenous antioxidant enzymes, including SOD, CAT, and GPx [17,18,20,21,22]. Moreover, several studies have shown that melatonin directly influences redox pathways, such as Nrf2, in various models of OS induction [23,24,25,26,27,28] and FoxO3 [29,30]. 

K562 erythroleukemic cells are a good alternative to assess the crucial role of FoxO3 and Nrf2 in the metabolic maintenance of erythroid precursors, making it possible to analyze their modulation and the action of melatonin on such pathways, as well as its antioxidant capacity. Thus, the investigation of melatonin as an agent that can act to modulate the oxidative stress of cells such as erythroid is valid and promising, not only for a better understanding of the redox adaptation pathways, but also for the validation of melatonin as a therapeutic alternative in the treatment of hematological alterations with chronic oxidative and inflammatory conditions. 

## 2. Materials and Methods

### 2.1. Cell Culture 

K562 cells (chronic myelogenous leukemia—ATCC cataloge number CCL-243) were maintained in RPMI-1640 (Roswell Park Memorial Institute, Buffalo, NY, USA) with 10% Bovine Fetal Serum (BFS, Hyclone, Logan, UT, USA), 100 U/mL penicillin, and 100 μg/mL streptomycin, and 0.25 μg/mL fungicide amphotericin B and incubated at 37 °C under a humidified atmosphere of 5% CO_2_. The cells were treated with 50 μM of hemin and 100 μM of hydroxyurea, following the protocol previously established to induce erythroid differentiation in the K562 cell line [31]. This study strictly complied with all Data Safety Monitoring Board (DSMB) standards for laboratory experimentation with cell culture, according to Brazilian Regulations.

### 2.2. Identification of Erythroid Differentiation and Viability

The erythroid differentiation of K562 cells was assessed by benzidine cytochemical test [32]. First, 50 µL of 30% hydrogen peroxide mixed with 10 µL of benzidine solution was added to 100 µL of cell suspension. The mixture was incubated in the dark, at room temperature, for 25 min and the percentage of benzidine-positive cells was determined by microscopic examination [31]. Cell viability was assessed using the Trypan blue (Sigma-Aldrich, St. Louis, MO, USA) exclusion assay [33], in the final concentration of 0.1%. The ratio between the number of living (L) and dead (D) cells provided the percentage (L/D × 100) of cell viability [33].

### 2.3. Experimental Design

The study had three pseudo-replicated samples followed during five days of cell differentiation, with collections of biological samples in three periods of the differentiation process: before the start (D0), at the beginning of differentiation (D2) and at the end of the process (D4). On day 0 of the experiment, 3.1 × 10^6^ cells/mL (100% viable and 70–80% confluent) were placed in polystyrene bottles in a final volume of 30 mL of culture, induced to erythroid differentiation through the addition of hemin (50 μM) and hydroxyurea (100 μM), and incubated at 37 °C/5% CO_2_. In addition, each sample was individually divided into the following groups: erythroid cells without inducing oxidative stress or melatonin treatment (Reference); cells under stress induction with hydrogen peroxide (100 µM H_2_O_2_); cells treated with a physiological concentration of 1 nM melatonin (C1) and a pharmacological concentration of 1 mM melatonin (C2); and two sets of cells treated with the same concentrations of melatonin associated with stress induction (C1 + H_2_O_2_ and C2 + H_2_O_2_, respectively).

### 2.4. Administration of Melatonin (Protective Agent) and Hydrogen Peroxide (Stressing Agent)

Treatments with low and high concentrations of melatonin (1 nM and 1 mM, respectively) were used. The concentration of 100 μM H_2_O_2_ was adopted, previously tested, and validated by de Paula (2020) for this experimental model [31]. The administrations of the antioxidant and peroxide were carried out daily and in a staggered manner in the associated groups (C1 + H_2_O_2_ and C2 + H_2_O_2_), that is, one hour after the administration of the respective concentrations of melatonin, H_2_O_2_ was added. The samples were collected 1 h after the administration of the stressor, on days 0 (D0), 2 (D2), and 4 (D4).

### 2.5. Real-Time PCR

RNA samples were subjected to DNAseI treatment (Invitrogen, Rockville, MD, USA) and reverse transcribed with a High Capacity cDNA Reverse Transcription kit (Thermo Scientific, Waltham, MA, USA) using oligo dTV and a random primer blend. The sequences of primers are shown in Table 1. The concentration of each primer was determined and the amplification efficiency was calculated according to the equation E^(1/slope)^ to confirm the accuracy and reproducibility of the reactions. Amplification specificity was by the dissociation protocol. qPCRs were performed in a StepOne Plus Real-time PCR System (Thermo Scientific), using SYBR Green GoTaq Master Mix (Promega, Madison, WI, USA). The fold change in mRNA level was calculated using 2^−∆∆Ct^ [34] and all the values were normalized to the expression of the beta actin (ACTB) gene. 

### 2.6. Statistical Analysis

Univariate analyzes were performed using the Statistica 9.0 software (Statsoft Inc., Tulsa, OK, USA), while the graphs were made using the GraphPad Prisma software version 5.01 for Windows (GraphPad Software, La Jolla, CA, USA). Data normality was verified using Normal Probability Plots of Residuals. Thus, some data were transformed by log10 when necessary. For comparison between groups, General Linear Models (GLM) were adopted in the ANOVA two-way format, allowing verifying the effects of treatments, incubation periods, as well as any interactions between these predictors on the dependent variables [35]. Each experiment was analyzed relative to its own reference group(s). The results were expressed as mean ± SEM of their biological values and *p* < 0.05 was statistically significant. We also developed a multivariate unsupervised analysis, a heatmap with hierarchical clustering only on the compounds. At the same time, samples were ordered based on their group labels using the web-based software for metabolomics, Metaboanalyst 5.0 (https://www.metaboanalyst.ca/ (accessed on 1 November 2022)) [36]. 

## 3. Results

### 3.1. Protective Role of Melatonin on Cell Viability

**Figure 1:**
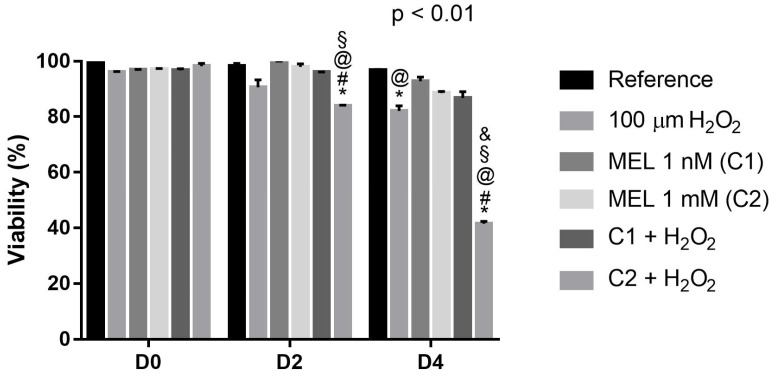
Viability of K562 cells followed for 5 days of culture with and without induction of oxidative stress by 100 μM of H_2_O_2_. Reference: K562 cells without induction of oxidative stress and not treated with melatonin; 100 µM H_2_O_2_: Cells under stress induction with hydrogen peroxide; C1: cells treated with 1 nM melatonin; C2: cells treated with 1 mM melatonin; C1 + 100µM H_2_O_2_ and C2 + 100 µM H_2_O_2_: sets of cells treated with the same melatonin concentrations associated with stress induction; D0: before the differentiation, D2: beginning of cell differentiation; D4: maximum of the differentiation process. * Effects of treatments within each period compared to the respective reference groups; ^#^ Effect of the incubation period within each treatment compared to their counterparts in D0; ^&^ Effect of the incubation period between D2 and D4 within the same treatment; ^§^ Effect of C1 + 100 µM H_2_O_2_ compared to treatment C2 + 100 µM H_2_O_2_ within the same period; ^@^ Effect of C2 compared to other treatments within the same period. Test performed: GLM with 2-way ANOVA design, complemented by Bonferroni test.

Regarding the viability of K562 cells, it is possible to observe that on day 0 (D0), under all conditions, the viability remained close to 100% (Figure 1). On day 2 (D2), treatment with the stressor agent caused a ~9% decrease in viability, even in the presence of the high concentration of melatonin tested (C2 + 100 µM H_2_O_2_), which (C2), in turn, did not show cytotoxic effects by itself. The low melatonin concentration associated with the stressor agent (C1 + 100 µM H_2_O_2_) maintained very similar viability to the reference, suggesting a cytoprotective effect. 

On day 4, we observed the same pattern of cell viability, but with an accentuation in the reduction of cell viability, particularly in the groups under the peroxide exposure, even in the presence of melatonin, especially the high dose (1 mM). Thus, once again, a possible cytoprotective effect was observed in the treatment using C1 + 100 µM H_2_O_2_. It is noteworthy that only the high melatonin concentration tested (C2) had an unexpected effect, mainly in the presence of the stressor agent (C2 + 100 µM H_2_O_2_–~20% and 60% decrease in viability on D2 and D4, respectively), suggesting a combined effect, triggering possible apoptotic pathways involved in this decrease in viability in this evaluated period.

### 3.2. FOXO3 and Regulators of Its Subcellular Localization

**Figure 2:**
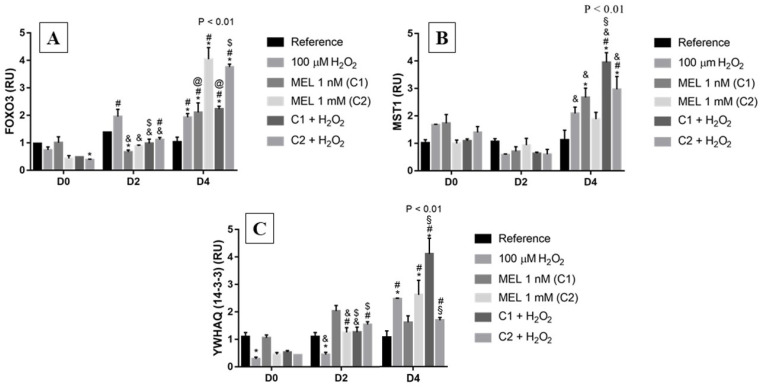
Relative expression of the *FOXO3* pathway in K562 erythroid cells: (**A**) *FOXO3* gene expression, (**B**) *MST1* gene expression, and (**C**) *YWHAQ* (14-3-3) gene expression. Reference: K562 cells without induction of oxidative stress and not treated with melatonin; 100 µM H_2_O_2_: Cells under stress induction with hydrogen peroxide; C1: cells treated with 1 nM melatonin; C2: cells treated with 1 mM melatonin; C1 + 100 µM H_2_O_2_ and C2 + 100 µM H_2_O_2_: sets of cells treated with the same melatonin concentrations associated with stress induction; D0: before the differentiation, D2: beginning of cell differentiation; D4: maximum of the differentiation process. * Effects of treatments within each period compared to the respective reference groups; # Effect of the incubation period within each treatment compared to their counterparts in D0; ^&^ Effect of the incubation period between D2 and D4 within the same treatment; ^§^ Effect of C1 compared to other treatments within the same period; ^@^ Effect of C2 compared to other treatments within the same period; ^$^ Effect of melatonin when compared to 100 µM H_2_O_2_. Test performed: GLM with 2-way ANOVA design, complemented by Bonferroni test.

Among the relative expressions, we observed that, as in most of the other genes analyzed, the expression of the references was constant regardless of the incubation period (Figure 2). For *FOXO3*, on day 0, C2 + H_2_O_2_ presented a lower expression when compared with their reference and were associated with a slight increase in *MST1* and a decrease in *YWHAQ* (coding gene for 14-3-3 protein). On D2, when under induction of oxidative stress with H_2_O_2_, we observed an increasing behavior in the expression of *FOXO3* (Figure 2A), with a 2-fold reduction in transcript level of *YWHAQ* (Figure 2C), differing from its counterpart on D0 and C1 + H_2_O_2_; unexpectedly, *MST1* (Figure 2B), also presented a decreasing behavior in transcript levels.

On D4, we observed an elevation in transcript levels of *FOXO3* under all treatments, with a 2-fold elevation when under H_2_O_2_ and the same pattern was also seen for *MST1* and *YWHAQ* (14-3-3). Moreover, for *FOXO3*, particularly on D4, it also presented different from its reference, as well as from treatments using 1 mM, with and without peroxide—treatments with the highest expressions on D4, which presented a 4-fold elevation in transcript levels; the same differences were observed for treatments with 1 nM (with and without peroxide, with a 2-fold elevation in gene expression) and in all treatments, *MST1* relative expression was higher than the reference, such as *FOXO3.*

### 3.3. Keap1/Nrf2/ARE Signaling Pathway

**Figure 3:**
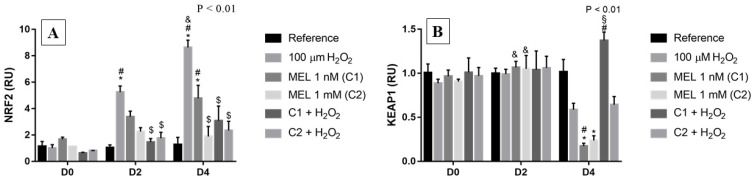
Relative expression of *NRF2* and of its regulator *KEAP1* in K562 erythroid cells: (**A**) *NRF2* gene expression and (**B**) *KEAP1* gene expression. Reference: K562 cells without induction of oxidative stress and not treated with melatonin; 100 µM H_2_O_2_: Cells under stress induction with hydrogen peroxide; C1: cells treated with 1 nM melatonin; C2: cells treated with 1 mM melatonin; C1 + 100 µM H_2_O_2_ and C2 + 100 µM H_2_O_2_: sets of cells treated with the same melatonin concentrations associated with stress induction; D0: before the differentiation, D2: beginning of cell differentiation; D4: maximum of the differentiation process. * Effects of treatments within each period compared to the respective reference groups; ^#^ Effect of the incubation period within each treatment compared to their counterparts in D0; ^&^ Effect of the incubation period between D2 and D4 within the same treatment; ^§^ Effect of C1 compared to other treatments within the same period; ^$^ Protective effect of melatonin. Test performed: GLM with 2-way ANOVA design, complemented by Bonferroni test.

For the Nrf2 pathway, we can observe that on D0 there was no difference in the expression between the reference and treatment with only the stressor agent (Figure 3A). However, on D2 and D4, there were 6- and 8-fold increases, respectively, in relative expression in the treatment with hydrogen peroxide alone compared with the reference groups; on D4 this increase was associated with a reduction on *KEAP1* expression when under H_2_O_2_ treatment (Figure 3B). Additionally, in the cells from days 2 and 4, we can observe a suggestive protective effect in the groups treated with melatonin under stress induction (C1 + H_2_O_2_ and C2 + H_2_O_2_), when comparing them to the 100 μM H_2_O_2_ group, maintaining Nrf2 transcript levels similar to those observed in the respective reference groups.

Furthermore, among the treatments using melatonin, it is observed that C1 showed the highest levels of transcripts in all analyzed periods, with a ~4-fold increase on D2 and D4. It is important to emphasize that, contrary to what we observed for *FOXO3*, there were no adverse effects suggestive of treatments with melatonin in any condition tested. Regarding *KEAP1*, on day 0 and 2, its expression remained stable regardless of treatment (Figure 3B). Finally, on D4, we observed a reduction in expression when C1 and C2 were used, which were different from their reference, the counterpart on D0, as well as the same treatments on day 2. 

### 3.4. Peroxiredoxins (PRDX)

**Figure 4:**
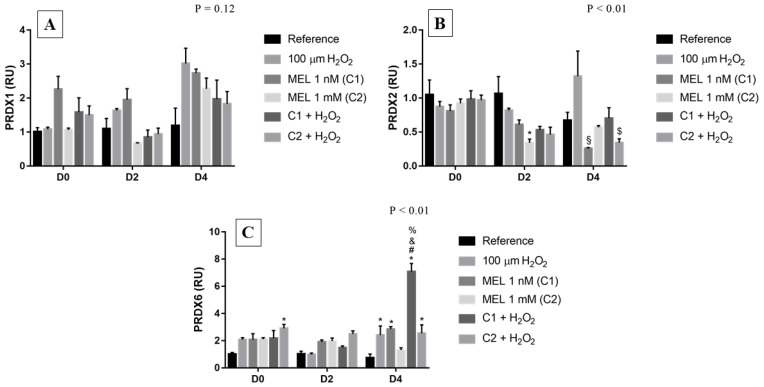
Relative expression of *PRDXs* in K562 erythroid cells: (**A**) Peroxiredoxin 1 (*PRDX1*) gene expression, (**B**) Peroxiredoxin 2 (*PRDX2*) gene expression, and (**C**) Peroxiredoxin 6 (*PRDX6*) gene expression. Reference: K562 cells without induction of oxidative stress and not treated with melatonin; 100 µM H_2_O_2_: Cells under stress induction with hydrogen peroxide; C1: cells treated with 1 nM melatonin; C2: cells treated with 1 mM melatonin; C1 + 100 µM H_2_O_2_ and C2 + 100 µM H_2_O_2_: sets of cells treated with the same melatonin concentrations associated with stress induction; D0: before the differentiation, D2: beginning of cell differentiation; D4: maximum of the differentiation process. * Effects of treatments within each period compared to the respective reference groups; ^#^ Effect of the incubation period within each treatment compared to their counterparts in D0; ^&^ Effect of the incubation period between D2 and D4 within the same treatment; ^§^ Effect of C1 compared to peroxide treatment within the same period; ^$^ Effect of melatonin; ^%^ Effect of treatment within the same period; Test performed: GLM with 2-way ANOVA design, complemented by Bonferroni test.

*PRDX1*, although not statistically significant, on all days, among the melatonin treatments, C1 treatment presented a ~2-fold increase in transcript levels when compared to its reference (Figure 4A); while the transcript levels in the other melatonin treatments on D2 and 4 were lower than in the groups treated only with hydrogen peroxide, particularly those under stress induction (C1 + H_2_O_2_ e C2 + H_2_O_2_). Moreover, on day 2 and 4, the highest gene expression of this enzyme occurred in the group under stress induction (100 μM H_2_O_2_), reflecting its importance to maintain the redox homeostasis of the cell. 

Regarding *PRDX6*, on D0 there was a ~2-fold increase, although not significant in most cases compared to the reference, in the expression of mRNA of this enzyme (Figure 4C). On D4, we observed an expressive increase in the levels of transcripts, being greater in the 1 nM treatment, mainly associated with stress induction (~7-fold increase), when compared with the reference. Surprisingly, in relation to *PRDX2*, the gene encoding the third most abundant protein in erythrocytes responsible for protecting them from ROS-mediated DNA damage during erythropoiesis [37], did not show high mRNA expression (Figure 4B). On D2 and D4, treatments with melatonin showed a pattern of inhibition of *PRDX2* expression when compared to reference groups and groups treated with the stressor agent. Only the C1 + H_2_O_2_ treatment, on D4, kept transcript levels more similar to the physiological than to the treatment with the stressor agent alone, thus suggesting a possible protective effect of melatonin. 

### 3.5. CAT, SOD1 and GPX1

**Figure 5:**
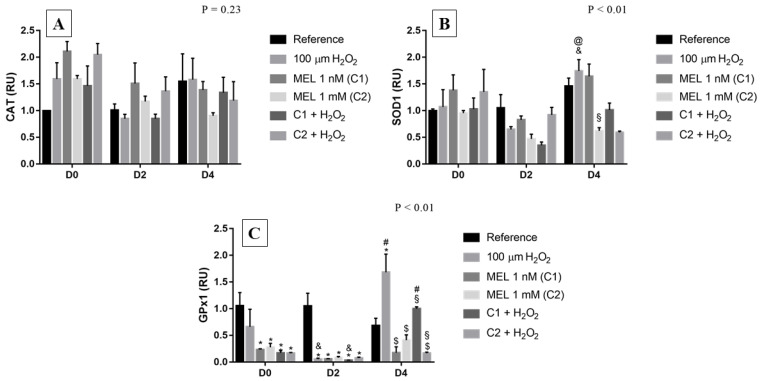
Relative expression of *CAT*, *SOD1,* and *GPx1* in K562 erythroid cells: (**A**) *CAT* gene expression, (**B**) *SOD1* gene expression, and (**C**) *GPx1* gene expression. Reference: K562 cells without induction of oxidative stress and not treated with melatonin; 100 µM H_2_O_2_: Cells under stress induction with hydrogen peroxide; C1: cells treated with 1 nM melatonin; C2: cells treated with 1 mM melatonin; C1 + 100 µM H_2_O_2_ and C2 + 100 µM H_2_O_2_: sets of cells treated with the same melatonin concentrations associated with stress induction; D0: before the differentiation, D2: beginning of cell differentiation; D4: maximum of the differentiation process. * Effects of treatments within each period compared to the respective reference groups; ^#^ Effect of the incubation period within each treatment compared to their counterparts in D0; ^&^ Effect of the incubation period between D2 and D4 within the same treatment; ^§^ Effect of C1 compared to other treatments within the same period; ^@^ Effect of C2 compared to other treatments within the same period; ^$^ Effect of melatonin; Test performed: GLM with 2-way ANOVA design, complemented by Bonferroni test.

Although not statistically significant, we can observe a clear induction behavior of gene expression of *CAT* on D0 when under hydrogen peroxide treatment (Figure 5A). Furthermore, on D0 and D2, an increasing behavior in expression was observed when under treatment with 1 nM compared with the reference; on D4, the expression pattern remained similar in all treatments, except for C2. As for *SOD1*, on day 0, the relative expression of this gene remained close to 1, showing a small increase when under C1 and C2 + H_2_O_2_ treatment (Figure 5B). Concerning D2 cells, there was a small reduction in transcript levels under all treatments used. On D4, however, a slight increase in transcripts levels occurred when under treatment only with the stressor agent, which is different from the same treatment in D2 and C2 with and without hydrogen peroxide. Among the treatments using melatonin, both on D0 and D4, C1 showed higher levels of transcripts compared with the reference. 

Unexpectedly, *GPx1*, in general, showed lower relative expressions in treatments with melatonin than in the reference and in the condition with only hydrogen peroxide (Figure 5C). On D2, there was a drastic reduction in the expression under all treatments, except for the reference, which remained at 1. On D4, we observed a possible protective role of melatonin when in the C1 + H_2_O_2_ treatment, since transcript levels were more similar to the reference than to the stress-induced group.

### 3.6. Proteasome Subunits: PSMB5 and PSMB6

**Figure 6:**
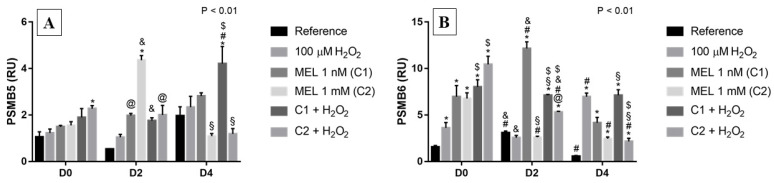
Relative expression of proteasome subunits *PSMB5* and *PSMB6* in K562 erythroid cells: (**A**) *PSMB5* gene expression and (**B**) *PSMB6* gene expression. Reference: K562 cells without induction of oxidative stress and not treated with melatonin; 100 µM H_2_O_2_: Cells under stress induction with hydrogen peroxide; C1: cells treated with 1 nM melatonin; C2: cells treated with 1 mM melatonin; C1 + 100 µM H_2_O_2_ and C2 + 100 µM H_2_O_2_: sets of cells treated with the same melatonin concentrations associated with stress induction; D0: before the differentiation, D2: beginning of cell differentiation; D4: maximum of the differentiation process. * Effects of treatments within each period compared to the respective reference groups; # Effect of the incubation period within each treatment compared to their counterparts in D0; ^&^ Effect of the incubation period between D2 and D4 within the same treatment; ^§^ Effect of C1 compared to other treatments within the same period; ^@^ Effect of C2 compared to other treatments within the same period; ^$^ Effect of melatonin when compared to 100 µM H_2_O_2_. Test performed: GLM with 2-way ANOVA design, complemented by Bonferroni test.

*PSMB5*, on day 0, presented a pattern of gradual increase according to the treatments used, with the highest expression observed for the C2 + H_2_O_2_ treatment, differing from the respective reference (Figure 6A). On D2, C2 presented the highest relative expression, with a 4-fold increase in expression when compared with its reference and the same treatment on D4, and also presented a 2-fold increase compared with C1 and C2 + H_2_O_2_ on the same day (D2). On the last day (D4), C1 + H_2_O_2_ had the highest transcript levels, presenting a ~2-fold increase when compared with the group with only H_2_O_2_ and a 4-fold increase compared with C2 and C2 + H_2_O_2_, the latter two being the ones with the lowest relative expressions. 

Regarding *PSMB6*, on the first day (D0), all treatments caused a ~4- to 10-fold increase in the relative expression levels for the gene in question when compared with the reference (Figure 6B). On the second day (D2), we observed a significant 12-fold increase in C1 treatment, when compared with its reference, and cells only under H_2_O_2_ and C2, which presented very similar expression levels; moreover, C1 also presented higher expression than C1 + H_2_O_2_ and C2 + H_2_O_2_. On D4, a ~7-fold increase in gene expression was observed when under treatment only with the stressor agent and C1 + H_2_O_2_, and a 4-fold increase in transcripts levels under C1 when compared to the reference.

### 3.7. BIM

**Figure 7:**
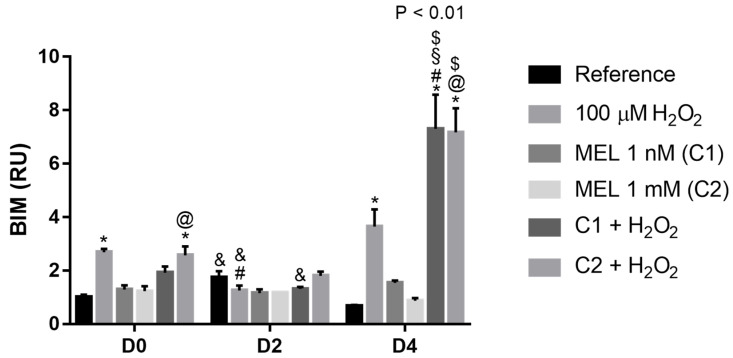
Relative expression of *BIM* in K562 erythroid cells. Reference: K562 cells without induction of oxidative stress and not treated with melatonin; 100 µM H_2_O_2_: Cells under stress induction with hydrogen peroxide; C1: cells treated with 1 nM melatonin; C2: cells treated with 1 mM melatonin; C1 + 100 µM H_2_O_2_ and C2 + 100 µM H_2_O_2_: sets of cells treated with the same melatonin concentrations associated with stress induction; D0: before the differentiation, D2: beginning of cell differentiation; D4: maximum of the differentiation process. * Effects of treatments within each period compared to the respective reference groups; # Effect of the incubation period within each treatment compared to their counterparts in D0; ^&^ Effect of the incubation period between D2 and D4 within the same treatment; ^§^ Effect of C1 compared to other treatments within the same period; ^@^ Effect of C2 compared to other treatments within the same period; ^$^ Effect of melatonin when compared to 100 µM H_2_O_2_. Test performed: GLM with 2-way ANOVA design, complemented by Bonferroni test.

For this gene that encodes a pro-apoptotic protein, we observed all cases where there was an increase in its expression occurred in the presence of the stressor agent (H_2_O_2_, C1 + H_2_O_2_ and C2 + H_2_O_2_) (Figure 7). It demonstrates that the stressor contributes to the increased stimulus to apoptosis in these cells, mostly when under C2 + H_2_O_2_ treatment, as can be observed, e.g., on D4 with a ~7-fold increase in gene expression. 

### 3.8. Overview of the Expression Pattern of the Genes Involved in the Redox Adaptation Mechanisms in K562 Cells

**Figure 8:**
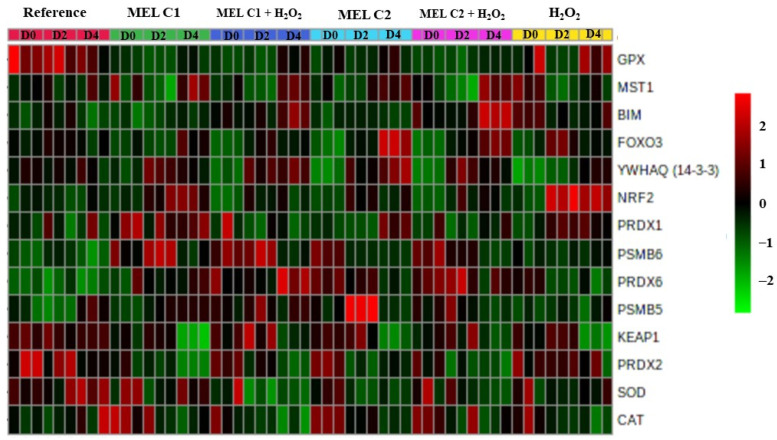
Overview of the expression pattern of the genes involved in the redox adaptation mechanisms in K562 cells. Genes are clustered hierarchically (full clustering method) using Euclidean correlation as the distance metric, with colors ranging from green (lowest) to red (highest) indicating the level of gene expression in each treatment and period evaluated. Reference: K562 cells without induction of oxidative stress and not treated with melatonin; 100 µM H_2_O_2_: Cells under stress induction with hydrogen peroxide; C1: cells treated with 1 nM melatonin; C2: cells treated with 1 mM melatonin; C1 + 100 µM H_2_O_2_ and C2 + 100 µM H_2_O_2_: sets of cells treated with the same melatonin concentrations associated with stress induction; D0: before the differentiation, D2: beginning of cell differentiation; D4: maximum of the differentiation process.

We created a heatmap to improve the overview visualization of our data set. Thus, Figure 8 summarizes the transcription level changes observed in our work, with colors ranging from green (lowest) to red (highest) indicating the level of gene expression in each treatment and period evaluated.

## 4. Discussion

The role of melatonin in the regulation of the antioxidant defense system and its protective effects against oxidative insults have been identified in several in vivo and in vitro studies [38,39]. In the latter, in models of oxidative stress induced by H_2_O_2_, both the suspension of peripheral blood mononuclear cells [40] and erythrocytes [41] occurred, showing that melatonin can prevent the undesirable effects of oxidative stress induced by H_2_O_2_. Normally, such results are associated with the continuous protection exerted by melatonin, through its antioxidant cascade [42]. On the other hand, in most cancer cells, in vitro, melatonin stimulates the production of endogenous ROS with consequent DNA damage and cell death [22]. Given the above, the present study, as far as we know, produced a unique opportunity in which, apparently, these modulatory effects of melatonin were found and associated with important redox adaptation pathways, in a model of K562 erythroleukemic cells induced or not by oxidative stress by H_2_O_2_.

Melatonin directly eliminates H_2_O_2_ and the metabolites (*N*^1^-acetyl-*N*^2^-formyl-5-methoxykynuramine—AFMK, then converted into *N*^1^-acetyl-5-methoxykynuramine—AMK) from this reaction still have a strong capacity for scavenging free radicals; in fact, the latter metabolite, in aqueous solution, reacts faster than melatonin with all free radicals [42]. This interaction of melatonin with H_2_O_2_ seems to exhibit two distinct phases: in the fast reaction phase, the action of melatonin on H_2_O_2_ (2.3 × 10^6^ M^−1^ s^−1^) forms AFMK, while in the slow phase there is a synergistic effect of melatonin and AFMK in the elimination of H_2_O_2_. Thus, it is estimated that about 2–5% of melatonin participates in the rapid reaction phase, when this occurs at a molecular ratio of 1:1 [43]. Therefore, it is plausible to hypothesize that both concentrations of melatonin tested had a direct detoxifying action on the stressor. 

The ability to adjust to oxidative stress (e.g., H_2_O_2_) through transitory modifications in gene expression is crucial for cellular defense mechanisms. In our study, melatonin had a relevant cytoprotective effect on differentiation-induced cells subjected to stress induction, at both tested concentrations, through the Nrf2-ARE redox signaling pathway regulation. This effect was demonstrated by the maintenance of transcript levels of this important TF similar to the respective reference groups (physiological levels), even under stress induction with peroxide, when compared to groups under the action of only the stressor agent (H_2_O_2_). This result suggested a direct-action effect of melatonin and its metabolites in the detoxification of H_2_O_2_, as well as other ROS formed in the experimental model studied, which culminated in the maintenance of similar levels to the physiological transcripts of some of the evaluated antioxidants.

As previously mentioned, Nrf2 is a key protein responsible for inducing the expression of antioxidant enzymes and, consequently, for maintaining cellular redox homeostasis. Thus, therapeutic alternatives that enable its regulation are extremely valuable, since this TF can modulate expression levels of hundreds of genes involved in combating OS and related pathophysiological states [44,45,46]. One of the agents with therapeutic potential that stands out is melatonin. Its indirect antioxidant effects on this redox pathway were related to a pattern of induction of *NRF2* expression when at a concentration of 1 nM, possibly causing an increase in the protein level, allowing its role in inducing the transcription of other genes. Such induction patterns of antioxidant genes could be observed, for example, in cells under treatment C1 on day 0, for *CAT*, *SOD*, *PRDX1*, and *6*. On day 2, the same pattern was observed for *CAT*, *PRDX1*, and *6*. Lastly, on D4, we observed this pattern for *SOD*, *PRDX6*, and *PRDX1*, even suggesting a protective effect of melatonin concerning the latter peroxiredoxin, since treatments using melatonin blunted the effects caused by treatment with peroxide, without significant cytotoxic events. Thus, our data corroborate that melatonin and possibly its metabolites have a variety of physiological and metabolic advantages that can increase their capacity to limit oxidative stress, in a period and dose-dependent manner in the studied model. 

It has already been reported that Nrf2 can be activated by proteasome inhibition [47,48]. This gene expression induction observed in our work may be explained by the fact that melatonin can act as a proteasome inhibitor [13,49], thus allowing the maintenance of higher levels of this TF acting in the nucleus, which could help in a consequent increase in the expression of its target genes, including antioxidant enzymes and 20S proteasome beta subunits. Previous studies demonstrated that indirect antioxidants, e.g., 3H-1,2-dithiole-3-thione (D3T), that as melatonin, induces many cellular antioxidants and phase 2 enzymes, increase expression of *PSMB5* and *PSMB6* proteasome subunits through the Keap1-Nrf2-ARE signaling pathway [15,16], with increased cellular resistance to oxidative stress [50]. Similarly, our data suggest that an Nrf2-dependent increase in proteasome subunit expression may be involved when under C1 treatment. 

Moreover, we observed a great increase in *NRF2* expression when the stressor agent was administered alone. Results by Pickering et al., using MEF cells, demonstrated that a mild dose of H_2_O_2_ caused a 2-fold increase in cellular Nrf2 levels, associated with increased nuclear localization of this TF [50]. The group also showed that under H_2_O_2_ exposure there is a strong increase in Nrf2 binding to ARE sequences on genes encoding many 20S proteasome subunits [50], in accordance with our results. However, it is noteworthy that there may be overlapping pathways of signal transduction that act synergistically, or antagonistically, adjusting proteasome levels in a very dynamic way [50]. 

Regarding the *FOXO3* gene, on the other hand, the same pattern suggestive of the protective effect of melatonin treatment on redox metabolism was not found. The maintenance of the expression of this gene in the reference throughout the evaluated periods may be associated with the role of this TF in cell proliferation and differentiation in the hematopoietic lineage [51], evidenced by the high viability in all periods. However, the addition of melatonin, especially at the high concentration associated with stress induction (C2 + H_2_O_2_) resulted in an increased expression of *FOXO3*, whose signaling pathway is possibly related to the induction of apoptotic pathways [29]. This possible activation of apoptotic pathways (day 4) at the expense of antioxidant defense pathways may be related to the characteristics of this biological model, as well as to a possible antioxidant stress triggered by the high reducing capacity of melatonin and its metabolites (AMFK and AMK), although there is still no clear evidence of this mechanism in vivo.

In this context, we can propose that melatonin at the high concentration associated with induction of oxidative stress interferes with the FoxO3 signaling pathway and probably its participation may have influenced the induction of apoptosis, especially in the last day and/or in the cell proliferation process regulated by FoxO3. Carbajo-Pescador et al. (2013) observed that treatment with melatonin (1 mM) increased FoxO3 activity with values that represented approximately 150% of the control after 48 h and caused apoptosis induction in HepG2 cells. This pro-apoptotic effect was demonstrated by the reduction of FoxO3 phosphorylation in Thr32 and Ser253, and induction of its increased nuclear localization through the action of melatonin, allowing this TF to act by increasing the expression of the Bim protein, causing apoptosis of these cells of cancerous origin [29]. Therefore, we tested whether *BIM* expression was elevated in the treatments that presented lower viability, demonstrating that in K562 this elevation might also be in a FoxO3-dependent manner via Bim. 

Thus, although the K562 lineage is widely used as a biological model for the study of cells with erythroid characteristics, it is still a tumoral lineage and, therefore, due to the antitumor action of melatonin, it is possible that despite the process of cell differentiation of K562 cells, activation of this apoptotic pathway occurred as an interesting cellular adaptive mechanism, allowing the survival of part of the culture in a compensatory way. That is, cells that were terminally compromised, possibly due to the effect of the antioxidant stress generated by the concentration of melatonin used plus the induction of oxidative damage by H_2_O_2_, were directed to cell death (apoptosis) as a way to protect the microenvironment for the cells that were still viable [52]. Moreover, it is worth mentioning that the members of this pathway are not exclusively related to *FOXO3*, they are also involved in wide range of processes. So far, for 14-3-3, it has been described that this chaperone is capable of interacting with more than 500 proteins, which have multiple functions in various cellular processes, such as signal transduction, apoptosis, cell cycle regulation and transcription [53,54]. As for MST1, this important kinase is associated with regulatory mechanisms for many biological events, including cell growth, apoptosis, stress response and senescence [55]. Therefore, the relative expression patterns may not always correspond to *FOXO3* expression. 

Finally, it is challenging to clearly understand the effect of melatonin as it does not act alone, increasing the complexity of the molecule itself as well as the study, reflected by certain intriguing patterns of the results presented in this work whose observed effects probably do not occur in vivo. Thus, additional work that contemplates the investigation of these important redox adaptation pathways associated with apoptotic pathways, with different concentrations of melatonin, as well as its metabolites, and in different experimental models, is still needed to understand and establish the mechanisms of action of melatonin on these pathways and which characteristics resulted from the experimental model studied.

## 5. Conclusions

Some limitations of the present study need to be acknowledged, such as the evaluation of post-translational modifications of Nrf2 and FoxO3, as well as the protein levels of the biomarkers. Notwithstanding, our experiments showed the effects of melatonin administration in erythroleukemic cells presented a period and dose-dependent pattern against the oxidative stress induced by H_2_O_2_, with direct and indirect action on the detoxification of the stressor agent, since the FoxO3 pathway presented a suggestive role of induction of apoptotic pathways, indicated by the elevation in *BIM* expression, while the transcription factor was predominantly responsible for the maintenance of redox homeostasis in K562 cells was Nrf2. Thus, the present work reinforces the importance of this indolamine in the regulation of cellular homeostasis, being a promising therapeutic alternative for diseases that present an exacerbated oxidative damage, due to its potent reducing effect.

## Figures and Tables

**Table 1:** Primers sequences.

Genes	Forward Primer	Reverse Primer
*ACTB*	5′-CAAGCAGGAGTATGACGAGTC-3′	5′-GCCATGCCAATCTCATCTTG-3′
*PRDX1*	5′-TGTAAATGACCTCCCTGTTGG-3′	5′-TATCACTGCCAGGTTTCCAG-3′
*PRDX2*	5′-CTGTTAATGATTTGCCTGTGGG-3′	5′-TGGGCTTAATCGTGTCACTG-3′
*PRDX6*	5′-CACGACTTTCTGGGAGACT-3′	5′-GGGCAATCAACTTAACATTCCTC-3′
*CAT*	5′-TGAATGAGGAACAGAGGAAACG-3′	5′-GTACTTGTCCAGAAGAGCCTG-3′
*SOD1*	5′-GGGCAAAGGTGGAAATGAAG-3′	5′-CAGCTAGCAGGATAACAGATGAG-3′
*GPX1*	5′-TTCCAGACCATTGACATCGAG-3′	5’-CACCCTCATAGATGAAAACCCC-3′
*FOXO3*	5′-GCGTGCCCTACTTCAAGGATAAG-3′	5′-GACCCGCATGAATCGACTATG-3′
*MST1*	5′-CCTCCCACATTCCGAAAACCA-3′	5′-GCACTCCTGACAAATGGGTG-3′
*YWHAQ (14-3-3)*	5′-GGGTTGCATCTCTTTCTTGC-3′	5′-GCACTCCTGACAAATGGGTG-3′
*NRF2*	5′-GCTACGTGATGAAGATGGAAAAC-3′	5′-AGCTCAGAAAAGGTCAAATCCTC-3′
*KEAP1*	5′-AACAGAGACGTGGACTTTCG-3′	5′-GTGTCTGTATCTGGGTCGTAAC-3′
*BIM*	5′-AACCACTATCTCAGTGCAAT-3′	5′-GGTCTTCGGCTGCTTGGTAA-3′
*PSMB5*	5′-CCATACCTGCTAGGCACCAT-3′	5′-GCACCTCCTGAGTAGGCATC-3′
*PSMB6*	5′-CCTATTCACGACCGCATTTT-3′	5′-TCCCGGTAGGTAGCATCAAC-3′

## Data Availability

The datasets generated during and/or analyzed during the current study are available from the corresponding author on reasonable request.
